# Maladaptive Daydreaming in a Psychiatry Outpatient Clinic: The Associations with Dissociation, Childhood Trauma and Emotional Dysregulation

**DOI:** 10.1192/j.eurpsy.2026.10446

**Published:** 2026-07-31

**Authors:** S. Karabulut, B. Başer Özkoç, O. Tosun

**Affiliations:** School of Medicine Psychiatry Clinic, Akdeniz University, Antalya, Türkiye

## Abstract

**Introduction:**

Maladaptive daydreaming (MD) is a distinct syndrome in which the main symptom is excessive vivid fantasising involving immersion in mental imagery of alternative realities that impairs functioning and causes significant distress. Although pathological immersive fantasising is a not a new clinical phenomenon, recently, there have been empirical and theoretical reasons for classifying it as a dissociative disorder.

**Objectives:**

In this study we aimed to determine the prevalance and clinical characteristics of MD in a sample of outpatients who admitted to the Akdeniz University outpatient clinic. We also aimed to compare MD related clinical variables between patients with and without dissociative disorders.

**Methods:**

A total of 165 outpatients were included in the study. Demographic and clinical data of the patients were recorded using a structured data form. All patients were evaluated with Dissociative Experiences Scale (DES), Maladaptive Daydreaming Scale (MDS), Difficulty in Emotion Regulation Scale (DERS) and Traumatic Experiences Checklist (TEC). The study was approved by the Akdeniz University School of Medicine Ethics Committee.

**Results:**

Out of the 165 patients, 28 had maladaptive daydreaming (16.9 %). Patients with MD were younger, had higher DES and DES-taxon scores, and DERS scores compared to those without MD (p<0.001). The rates of regular alcohol drinkers, deliberate self-harm behaviors and childhood emotional neglect were also higher in patients with MD. Among patients with DD, rate of MD was higher compared to those without DD (32.5 % vs 14.5 %, p=0.03). Linear regression analysis revealed that only DES scores predicted MDS scores (p<0.001; OR (95%CI) = 0.53 (0.40-0.75)).

**Conclusions:**

To the best of our knowledge, this is the first study reporting MD prevalance among individuals with DD, from Türkiye. Our results showed that there is a strong relationship between dissociation and maladaptive daydreaming. The two disorders co‐occur with each other and maladaptive daydreaming is strongly linked to dissociation in populations with high levels of trauma and emotional dysregulation. Clinicians should screen both symptoms when evaluating patients with childhood trauma. Despite its significant dissociative components, further research incorporating diverse diagnostic perspectives is warranted.

**Disclosure of Interest:**

None Declared

